# Emulsion Gels Formed by Electrostatic Interaction of Gelatine and Modified Corn Starch via pH Adjustments: Potential Fat Replacers in Meat Products

**DOI:** 10.3390/gels9010050

**Published:** 2023-01-07

**Authors:** Abu Bakar Asyrul-Izhar, Jamilah Bakar, Awis Qurni Sazili, Yong Meng Goh, Mohammad Rashedi Ismail-Fitry

**Affiliations:** 1 Department of Food Technology, Faculty of Food Science and Technology, Universiti Putra Malaysia, Serdang 43400, Selangor, Malaysia; 2 Department of Animal Science, Faculty of Agriculture, Universiti Putra Malaysia, Serdang 43400, Selangor, Malaysia; 3 Halal Products Research Institute, Universiti Putra Malaysia, Serdang 43400, Selangor, Malaysia; 4 Department of Veterinary Preclinical Sciences, Faculty of Veterinary Medicine, Universiti Putra Malaysia, Serdang 43400, Selangor, Malaysia

**Keywords:** canola oil, electrostatic complexes, emulsion gel, fat analogue, fat replacers, microstructure, protein-polysaccharide interaction, X-ray diffraction

## Abstract

The application of emulsion gels as animal fat replacers in meat products has been focused on due to their unique physicochemical properties. The electrostatic interaction between proteins and polysaccharides could influence emulsion gel stability. This study aimed to evaluate the physicochemical properties of emulsion gels using starch and gelatin as stabilizers, promoting electrostatic attraction via pH adjustment. Three systems were studied: emulsion gel A (EGA) and emulsion gel B (EGB), which have positive and negative net charges that promote electrostatic interaction, and emulsion gel C (EGC), whose charge equals the isoelectric point and does not promote electrostatic interactions. There was no significant difference in proximate analysis, syneresis and thermal stability between samples, while EGA and EGB had higher pH values than EGC. The lightness (L*) value was higher in EGA and EGB, while the yellowness (b*) value was the highest in EGC. The smaller particle size (*p* < 0.05) in EGA and EGB also resulted in higher gel strength, hardness and oxidative stability. Microscopic images showed that EGA and EGB had a more uniform matrix structure. X-ray diffraction demonstrated that all the emulsion gels crystallized in a β′ polymorph form. Differential scanning calorimetry (DSC) revealed a single characteristic peak was detected in both the melting and cooling curves for all the emulsion gels, which indicated that the fat exists in a single polymorphic state. All emulsion gels presented a high amount of unsaturated fatty acids and reduced saturated fat by up to 11%. Therefore, the emulsion gels (EGA and EGB) that favored the electrostatic protein-polysaccharide interactions are suitable to be used as fat replacers in meat products.

## 1. Introduction

Recently, the public has shown more concern about health-related foods, and as a result, the demand for healthier meat products has increased [1,2]. Fat or lipid content in meat products could be reformulated to provide wholesome and functional foods, thus allowing consumers to choose healthier products [3]. Various strategies for reducing lipid content and improving the lipid profile of meat product formulations, which are mostly composed of back fat, a major source of saturated fat, have been proposed. Among the strategies included the use of polyunsaturated fatty acid-rich vegetable oils in liquid forms, dietary fibers, hydrocolloids, and, more recently, emulsion gels, hydrogels, and organogels [4,5,6]. The food industry and research community have paid close attention to edible oil structure as a possible solution to substitute saturated fats with healthier alternatives such as polyunsaturated oil [7]. Nevertheless, the application of these reformulations as a single strategy does not satisfy the desired technical and sensory features since instability in the meat matrix during the processing stages was detected, as well as having a negative effect on consumer approval [8,9]. Thus, understanding the interactions between different components and the methods of adding them to formulations is important for producing stable and approved fat-reduced meat products [5]. 

Food-grade biopolymers such as proteins and polysaccharides have been employed as structural agents in the oil structuring process [10,11]. The application of hydrocolloids for this purpose has some benefits because they are acknowledged and recognized by the food industry, are considered Generally Recognized as Safe (GRAS) by the Food and Drug Administration (FDA), avoid regulatory restrictions, and are easily accessed [7]. These associations of colloids are not subject to regulations and may offer textural and structural advantages [12]. Due to these factors, hydrocolloids have been employed to form systems that will substitute saturated fats, resulting in end products with properties identical to the original animal fat [13].

Modified starch is known to be such an effective fat replacer because some of the size globules have the same shapes and sizes as fat globules [14]. Moreover, modified starch also has distinctive physiochemical characteristics, which include viscous flow, water holding capacity, and gel formation, and can further reduce the calorie content of the food [15,16]. The presence of starch as a polysaccharide can impart a sense of creaminess to the mouth as a lot of water can be bound by it [17]. The modified starches can deliver lipophilic tastes and nutrients and combine with other food ingredients, such as proteins, to enhance the quality of low-fat products even further [18]. 

Gelatin is known to be an effective gelling agent and protein-based fat replacement due to its potential to mimic the smoothness and lubricity characteristics of fat [19]. The most important characteristic of gelatin is its capacity to form thermo-reversible gels, as when the temperature increases, the gelation gel dissolves, thus providing unique “melt-in-mouth” characteristics [20,21]. This makes gelatin an important texturing and flavor-releasing food ingredient [22]. Gelatin also functions as a good coating by forming a protective colloid and film, besides receiving a lot of attention as a foundation for the development of electrostatic compounds [19,23]. As a result, protein-polysaccharide complexes have been developed as fat replacers, particularly for use in processed meat [24].

In recent years, the oil structuring method, known as emulsion gel, has received a lot of attention as it is utilized as a fat replacer to replace animal fat in meat products. An emulsion gel resembles a gel-like solid material with a spatial network structure created based on an emulsion using a specific induction method. It is identified by the presence of emulsified oil droplets in the gel network [25,26]. An interesting approach to the development of emulsion gels would be the use of different types of proteins or polysaccharides. It is worth noting that emulsion gels made solely with protein or polysaccharide have significant practical constraints, such as poor emulsification and thermal stability [27]. This has gradually shifted the research focus to emulsion gels stabilized by several ingredients [28,29]. This can not only improve individual material emulsification but also help to improve the stability of emulsion gels [30,31]. 

Protein-polysaccharide interactions are also involved in a wide range of activities that alter the mechanical, sensory and functional aspects of food products, such as an emulsion gel [32]. Physical stability and textural properties of colloidal systems in food manufacturing can be enhanced using well-established and effective ways of associative interactions between proteins and polysaccharides [33]. These types of macromolecules can interact in a wide range of ways, including non-covalent attraction forces such as electrostatics, hydrogen bonding, hydrophobicity and van der Waals interactions [34]. A physicochemical phenomenon known as electrostatic complexation occurs when two polymers with opposing charges come into contact with one another due to electrostatic attraction, resulting in phase separation into a polymer-rich phase and a polymer-depleted phase [35]. The resulting protein-polysaccharide electrostatic interactions can combine the features of the biopolymers that make them up while also exhibiting novel structural and functional capabilities not found in their parent biopolymers [36]. As a result, electrostatic complexation may be appropriate for developing fat replacers to address the texture and flavor issues that are typically related to low-fat or low-carbohydrate diets [19]. 

Furthermore, with suitable treatment and modification, an emulsion gel with a denser and more homogeneous network structure can be created, altering the characteristics of the emulsion gel [27]. Extrinsic factors such as pH might influence electrostatic interactions between oppositely charged protein-coated droplets and polysaccharide molecules [37]. pH is a key component that influences the characteristics of emulsion gels (i.e., shape, structure, and gel strength) and the stability of lipids [37]. The pH of emulsions can influence not only the gelation characteristics but also the interactions between the proteins and polysaccharides. The mixture of these emulsions can be achieved by the dispersion of anionic polysaccharide/cationic protein complex dispersion at pH < pI, and emulsions can be formed by adding oil and homogenization [26]. Various studies have described the implementation of electrostatic complexes in the oil structuring application, i.e., electrostatic complexes of pectin and gelatin [38], soy protein/κ-carrageenan [39], whey protein isolate/pectin [40,41], gelatin/OSA starch [19] and modified starch/chitosan [42].

To the author’s knowledge, no work has previously been carried out to form electrostatic interactions using modified cornstarch and gelatin. The purpose of this study was therefore to characterize the formation and characteristics of electrostatic interactions formed from modified cornstarch and gelatin. Molecular composition, physicochemical properties and melting behaviors are characterized and their effects on mechanical properties are established. In particular, the characteristics of emulsion gels generated from these proteins and polysaccharide interactions may be altered in a controlled manner by modifying environmental conditions. A better knowledge of this type of emulsion gel might lead to its implementation in a variety of food items, such as meat products, with varying textural requirements.

## 2. Results and Discussion

### 2.1. Proximate Compositions 

Proximate analyses for three different emulsion gels showed no significant differences (*p* > 0.05) (Table 1). The moisture contents ranged from 57.14–58.95%. This data was reported to be around the range of emulsion gels prepared with olive oil and soy protein isolate [43]. The emulsion gels prepared with modified corn starch and canola oil have higher moisture contents compared to the other types of emulsion gels previously studied [1,44,45], because the modified corn starch itself has a high moisture content [46]. The usage of starches, especially modified starches, increased the moisture content due to their higher water absorption capacity [47]. The combination of proteins and polysaccharides also contributed to the higher moisture content of the emulsion gels. As a result, it is expected that a more diverse variety of phase behaviors could be achieved in a polysaccharide-protein combination. These phase behaviors might be caused not only by long- or short-range interactions between polysaccharides and proteins but also by differing affinities between the polymers and the solvent (water), thus leading to a higher moisture content of the emulsion gels [48]. The ash and protein were also not affected by the different treatments, ranging between 2.35–2.53% and 2.48–3.28%, respectively. The fat content of all the emulsion gels did not show any significant difference between treatments. The development of emulsion gels in this study with a fat content that ranged between 6.87–8.33% showed a relatively lower amount of fat content compared to the different types of animals that were usually used in the processing of meat products, as reported previously, which is as high as 31.44% [49,50]. The percentage of carbohydrates found in the present study showed values that varied between 27.87–30.20%.

### 2.2. pH Values

The final pH values of the emulsion gels were affected (*p* < 0.05) by varying the pH of the dispersion used to produce the emulsion gels. The pH of EGA and EGB were 5.03 and 4.5, respectively. These pH values were within the range described in emulsion gels stabilized with chia and cold gelling agents [1,51]. The highest (*p* < 0.05) pH values were observed in samples with electrostatic protein-polysaccharide interactions, and the lowest pH values (*p* < 0.05) were found in emulsion gels without electrostatic protein-polysaccharide interactions. EGA and EGB with high pH values are suitable to be applied in meat products as they could facilitate raising the pH of the meat system and increasing the water-holding capabilities by pushing the pH away from the isoelectric point [52], as the myofibrils loosen up as the charge moves away from the isoelectric point, which allows more water to penetrate the meat, increasing the water-holding capacity.

### 2.3. Color

Color is crucial in the development of fat analogues since it is one of the primary elements influencing consumer food choices, particularly meat products [1]. In terms of the color of the fat replacers, a lighter and less yellow fat color is more suitable for imitating animal fat, which is often seen in meat processing [53]. This study showed that EGC had a significantly (*p* < 0.05) lower lightness value and a higher yellowness value compared to EGA and EGB. This indicated the appearances of EGA and EGB were more suitable to be used as animal fat replacers in meat processing, with significant (*p* < 0.05) higher lightness and lower yellowness values. The redness value of EGA was comparable to the emulsion produced from perila and canola oil [53]. The color of the emulsion gels could be related to the particle size of the emulsion gels with different treatments. For instance, EGC, which did not have any electrostatic protein-polysaccharide interactions, had a significantly (*p* < 0.05) bigger particle size and showed a significant difference in the color values compared to EGA and EGB. The lightness value of oil-in-water emulsions decreased with increasing droplet size because the reflectance decreased as droplet diameter increased. After all, the scattering efficiency of the droplets was reduced, allowing the light beam to penetrate deeper into the emulsion and be absorbed more completely [54].

### 2.4. Syneresis and Thermal Stability

There was no noticeable syneresis with very little exudate release for all the samples of modified starch emulsion gel with different pH treatments. All samples showed less than 1% syneresis, with no significant difference (*p* < 0.05) between samples. Sato et al. [55] reported that emulsion gel formed with gelatin was tested stable for phase separation stability. Another study also found that heat treatment did not affect the release of chia or oat olive oil-in-water emulsion gels [1]. The water- and fat-binding capabilities during cooking are important for meat products. Since the samples showed no visible phase separation or degradation of the gel’s physical structure after heating, all the emulsion gels developed were thermally stable and could be used as fat replacers in meat products.

The behavior of the emulsion gels irrespective of the state (with or without electrostatic protein-polysaccharide interaction), their thermal stability (total and water loss) was less than 1%. The emulsion gels thus showed excellent thermal water binding properties despite containing a high proportion of water (around 58%). The high thermal stability of the emulsion gels can be attributed to the presence of modified corn starch and gelatin as the gelling agents. Both the protein and polysaccharides present in the emulsion gels improve the stability of emulsions with respect to pH [56]. Proteins, which are surface active, can stabilize the emulsions in the presence of polysaccharides by electrostatic interactions. Polysaccharides, on the other hand, because they are hydrophilic, often remain in the aqueous phase and control the aqueous phase rheology, such as thickening, gelling and functioning as stabilizing agents [57]. Even though there was no significant difference (*p* > 0.05) between each treatment of emulsion gels, EGC showed the highest amount of total and water loss compared to EGA and EGB due to the adjustment of the pH of EGC near its isoelectric points. The pH adjustment near the isoelectric pH (pI) of proteins and before heating (>60 °C) could cause droplet aggregation [58], which affects the emulsion stability.

On the other hand, EGA and EGB have lower amounts of total and water losses. Electrostatic interactions take place when oppositely charged biopolymers interact with one another [59], which is shown in the treatment of EGA and EGB. Protein molecules had a total positive charge at pH levels lower than the pI, acting as polycations. Anionic polysaccharides with carboxyl groups, in turn, operate as polyanions and can form electrostatic interactions in the presence of proteins. The presence of anionic branched polysaccharide with protein also enhanced the heat stability and inhibited the collision of droplets in a mixed emulsion [60]. The protein and polysaccharide interaction produced could withstand thermal treatment despite some alterations in its characteristics [59]. A similar study showed that the electrostatic interaction between protein and polysaccharide with opposite charges results in the formation of a stable compound [61].

### 2.5. Gel Strength and Hardness

Food mechanical properties, especially gel strength and hardness, influenced how consumers perceived their textural characteristics. In the case of meat products, the mechanical properties of fat play an important role. EGA had a significantly (*p* < 0.05) higher gel strength compared to EGB and EGC. This can be attributed to their effect on increased viscosity, which limited the mobility and collision frequency of the protein molecules, thereby slowing protein gelation, and to their role in steric hindrance, which interfered with the formation of protein-protein interactions, particularly disulfide bonds [62]. The smaller particle size of EGA also contributed towards a higher gel strength value. The formation of a larger number of smaller droplets would lead to a better-aggregated network of three-dimensional structures vital for gelation [63]. The gel strength of EGC was also lower than that of EGA and EGB as it had a larger droplet size. The 3D network of very attractive droplets (EGA and EGB), which is shown in the microscopic images of emulsion gels as opposed to random jamming of repulsive droplets (EGC), might explain such a significant improvement in gel strength [64]. The gel network’s strength was then also reduced due to a decrease in the filler effects of oil droplets as active fillers and subsequent interactions between oil droplets and proteins [65].

Further, by determining the emulsion gel hardness, we found that the gel hardness showed a changing trend similar to that of the gel strength. Table 2 indicated that when the pH of the emulsion gel favored the electrostatic protein-polysaccharide interactions (EGA and EGB), the gel hardness exhibited a significantly (*p* < 0.05) increasing trend. Variation of the emulsion droplet size might result in greater hardness, demonstrating that manipulating the droplet size could effectively regulate the gel’s texture [66]. This is similar to this study, which showed that EGC that has a larger (*p* > 0.05) droplet size has a lower hardness value, while EGA and EGB that have a smaller (*p* < 0.05) droplet size have a significantly (*p* < 0.05) higher hardness.

### 2.6. Particle Size and Zeta Potential

EGC showed a significantly (*p* < 0.05) larger particle size compared to EGA and EGB. The net charge in EGC, which was approaching the isoelectric point, caused the electrostatic repulsion forces between the droplets to be no longer strong enough to prevent droplets from drawing close together, resulting in flocculation [25], thus leading to larger droplet size. Significant agglomeration also occurs around the isoelectric point, with large flocs observed because the particle surface charge is close to zero and attractive van der Waals forces are dominant [67]. Significantly (*p* < 0.05) smaller droplet size was observed for EGA and EGB, which had oppositely charged proteins and polysaccharides, and more hydrophobic groups can come into contact with the canola oil, resulting in smaller droplet size [7]. The particle size is also smaller because the electrostatic repulsive force contributes to the prevention of agglomeration [67]. Further insights into the impacts of pH on the properties of the emulsions were obtained by measuring the zeta potential. The zeta potentials of EGA, EGB and EGC were −6.5 mV, +3.5 mV and +1.9 mV, respectively. The electrostatic charge that the oil droplets carry is a critical stability component for oil-in-water emulsions. Highly positively or negatively charged particles will resist one another in solutions, making flocculation less likely. This is proven by the microstructure of the emulsions in this study, which indicated that EGA and EGB have fewer particles flocculating with each other compared to EGC. Emulsions that have a high zeta potential, whether positive or negative, are electrically stabilized, whereas emulsions that have a low zeta potential tend to coagulate or flocculate, which might compromise their physical stability [68]. EGA also had a smaller particle size, and its high negative surface charges could stabilize the emulsion system by electrostatic repulsion, which is similar to a study by Lee et al. [69].

### 2.7. Lipid Oxidation

The oxidative stability of emulsions is greatly influenced by the viscosity [70] and pH [71] of the aqueous solutions. EGC presented significantly (*p* < 0.05) lower oxidation stability compared to EGA and EGB. The increased viscosity of the aqueous phase slows the transit and transfer of reactive species during oxidation, which protects PUFAs in gelled emulsions, thus improving oxidative stability [72]. The interactions between the biopolymers of protein and polysaccharide in the emulsion gels could contribute to a greater possibility for structure modifications through electrostatic interactions and assist in preventing PUFA oxidation in emulsions [73], thus higher oxidative stability was observed in both EGA and EGB compared to EGC. This complex protein-polysaccharide interaction was also able to inactivate peroxyl radicals in the emulsion, avoiding the development of secondary oxidation products [74]. The reduction in droplet size could also enhance the oxidative stability of an emulsion as it improved the gravity aqueous stability [75]. A similar finding was obtained in this study, where the particle size results (Table 3) indicated that EGA and EGB have a significantly (*p* < 0.05) smaller particle size than EGC, and therefore, EGA and EGB had higher oxidative stability compared to EGC.

### 2.8. Oil Binding Capacity

**Figure 1:**
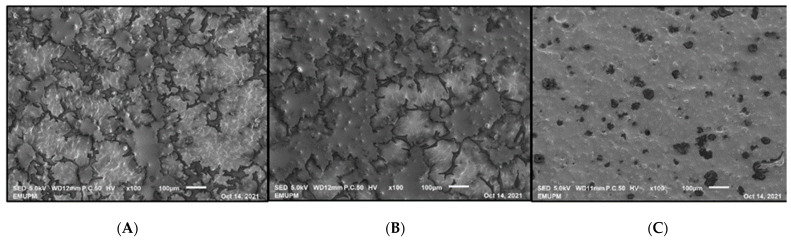
Microstructure images of (**A**) EGA, (**B**) EGB and (**C**) EGC.

The oil binding capacity values showed that EGA and EGB have a significantly (*p* < 0.05) higher oil-binding capacity compared to EGC. Nevertheless, considering these values, we can affirm that the oil binding capacity of all the formulated emulsion gels was adequate and suitable for their application in food matrices. For instance, Espert et al. [76] indicated that a great oil binding capacity is shown if it is approximately 90%. This high oil retention could be attributed to the fact that the presence of polysaccharides in emulsion gel provided higher oil retention, most likely due to the polysaccharide’s high capacity to form hydrogen bonds and hydrophobic interactions, which provided high stability at the interface and improved the final emulsion gel’s stability. EGC showed a significantly (*p* < 0.05) lower oil binding capacity, probably due to insufficient network formation to retain the oil droplets [11]. The microscopic image of EGC (Figure 1) showed that EGC had less network formation if compared to EGA and EGB.

### 2.9. Microstructure

Microscopic images of EGA and EGB developed a macroscopically three-dimensional network due to the absence of electrostatic repulsion between droplets [77]. EGA and EGB had a more uniform matrix structure compared to EGC. The electrostatic attraction presented in EGA and EGB was able to entrap the oil droplets in the gel network. The results for particle size also indicated that the oil droplets in EGC were much larger in size, and the microscopic images proved that they did not appear to homogeneously distribute within or be firmly bound to the gel network. This could be attributed to the pH alteration process, in which large fluctuations in pH reduced the emulsion stability, resulting in the destabilization of the emulsion and the coalescence of oil droplets, as well as fewer protein molecules absorbed at the oil/water surface [78].

### 2.10. X-ray Diffraction

**Figure 2:**
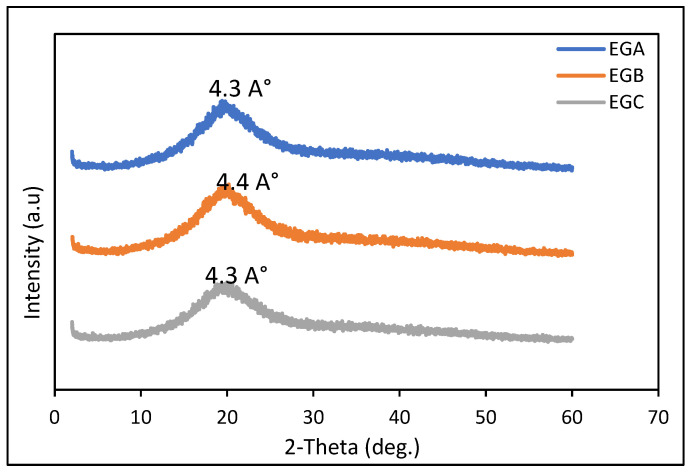
X-ray diffraction (XRD) patterns of EGA, EGB and EGC.

From a structural standpoint, edible fats are a subclass of soft matter that shares properties with materials of very different types. Fats are complex, heterogeneous mixes of triacylglycerols (TAGs) and TAG complexes with varying melting points. TAGs with higher melting points crystallize from the melt into various polymorphic forms, generating a crystal network that traps the liquid TAGs inside. Figure 2 shows the XRD spectra of emulsion gels with different treatments. All samples exhibited a broad diffraction peak. A wide peak indicated that the internal structures of the emulsion gels were similar. This might be owing to the polysaccharide molecular chain’s expansion and entanglement, resulting in a conformational transition [79,80]. The wide-angle region peaks for the EGA, EGB and EGC samples were identified at around 4.2–4.9 Å, 4.4–4.7 Å and 3.9–4.2 Å, respectively. The figure showed that these peaks were retained in all of the emulsion gels, and their intensity was not significantly changed, and the corresponding emulsion gels have similar polymorphic forms, which is the beta prime (β′) polymorphic form. This is probably due to the presence of monounsaturated fatty acids, which is shown in the fatty acid results of this study [81]. The dominant peak at 4.4 Å for EGB that shifted slightly to higher spacing values (normally 4.3 Å) indicates the presence of a (most probably small) fraction of beta prime (β′) polymorphic form. This also showed that the treatments on the emulsion gel did not affect the polymorphic forms of the emulsion gels. This formulation is suitable to be implemented as fat replacers in meat products, as the polymorphic form is similar to a study by Tiensa et al. [82], which indicated that higher quantities of the beta prime (β′) polymorphic form were observed in pates and extracted fat samples. Lard, which is usually used as a source of fat in meat products, also has the β′ polymorph, which is smoother and has a superior mouth feel [83]. The discovered XRD diffraction peaks for emulsion gel samples were identical to the peaks of the β′ polymorphic form of triglyceride crystals, according to prior research [84].

### 2.11. Fatty Acids Profile

Due to the current increased demand for a healthy diet, reducing animal fat while also modifying the fatty acid composition of meat products has become an emerging strategy. Table 4 showed the fatty acid composition of the emulsion gels and their distribution with regard to nutritional ratios. All emulsion gels registered the highest unsaturated fatty acid content compared to saturated fatty acids. The total amount of saturated fatty acids was decreased to levels below 11% of total fatty acids when emulsion gels were formulated as a fat replacer in meat products. Oleic acid was the most abundant fatty acid in all samples, which gave health advantages as well as possible applications in meat products [53], and this was due to the incorporation of canola oil in the emulsion gel formulation. In terms of polyunsaturated fatty acids, linoleic acid was present in the highest amount of all these emulsion gels, which had a positive and relevant effect on human health [85]. This effect was consistent with the fact that canola oil contains high levels of linoleic acid [86]. This was similar to a study by Pintado et al. [87], which indicated that regardless of the incorporation approach, meat products using chia flour and olive oil had the lowest saturated fatty acids and the highest monounsaturated fatty acids (mostly oleic acid) among the reduced fat samples and exhibited similar polyunsaturated (linolenic acid) values that were more than three times greater than any of the animal fat samples.

### 2.12. Differential Scanning Calorimetry

**Figure 3:**
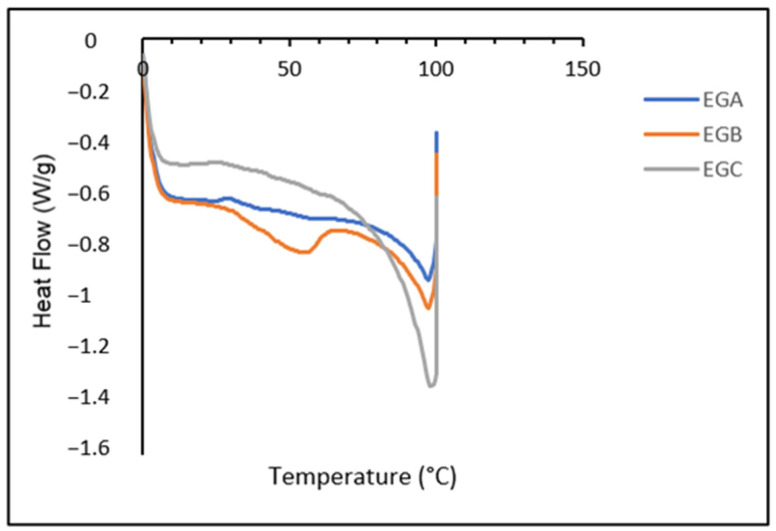
Differential scanning calorimetry (DSC) melting curve of EGA, EGB and EGC.

The melting profiles of the emulsion gels are shown in Figure 3. Three different types of emulsion gels resulted in a wide and single melting peak. The DSC analysis also showed that the alteration in pH of the emulsion gels did not alter the melting profiles of the emulsion gels. Instead, high melting points were visible for all emulsion gel samples, with the main peak centered at around 96 °C. The addition of hydrocolloids (modified corn starch and gelatin) in the formulation of emulsion gels contributed to the high melting point of the emulsion gels. Yilmaz et al. [88] indicated that the addition of hydrocolloids causes a significant increase in the peak values of the low-fat meat emulsions. Fat analogues formulated with potato starch exhibited high meltability due to the lack of thermoplasticity in starch [89]. Bi et al. [90] also discovered that starch significantly impeded meltability. Samples containing wheat starch also did not exhibit melting behavior upon heating [91]. The addition of starch also affects the water content of oleogels, which results in the endothermic peak of oleogels moving to a higher temperature [92].

A single endothermic transition corresponding to ice melting, consistent with a moisture content averaging ~59% in the encapsulated samples. DSC traces at high temperatures were flat, indicating that current emulsion gels were thermally stable at least up to 100 °C. This could also be attributed to the evaporation of the bound water and indicated the emulsion gel’s denaturation temperature, suggesting that all the emulsion gels had good thermal stability [93]. It has also been proposed that this single endothermic peak represents the development of thermodynamically more stable crystals [94]. The protein-polysaccharide composition of the emulsion gels also contributes to the ability of gel-like processed food items to withstand heat treatments [57]. All these thermal data suggested that the replacement of animal fats in meat compositions with emulsion gels may be favorable in terms of processing energy consumption [95].

**Figure 4:**
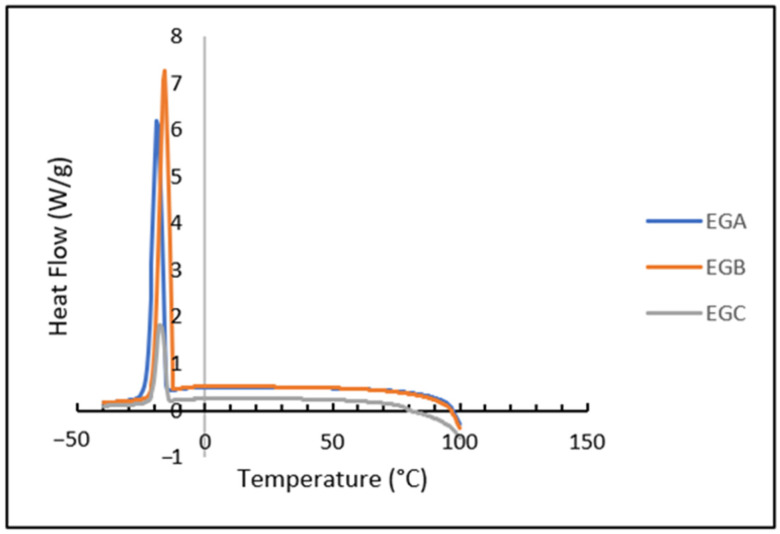
Differential scanning calorimetry (DSC) cooling curve of EGA, EGB and EGC.

The cooling profile of all the samples showed a sharp exothermic peak, indicating that the initial lipid nucleation and crystal formation were completed within a short time [96]. Figure 4 showed that the increase in the cooling profiles of all the emulsion gels, which ranged from −11 °C to −16 °C, showed that the rise in the crystallization temperature of dispersed oil in an emulsion solution was a typical effect of the emulsifier [97]. The exothermic peak also depicted that the cooling and freezing processes gradually increased from EGC to EGB and EGA. This was similar to the results of the exothermic reaction of soy protein gels [98]. The formulation of the emulsion gels, which incorporated polysaccharides and proteins, acts as an emulsifier, which in general has both hydrophobic and hydrophilic areas. The hydrophilic group of emulsifier molecules was oriented towards the aqueous phase, whereas the hydrophobic groups were oriented towards the oil phase. The predominant hydrophobic groups in an emulsifier are fatty acids, which were then aligned in a complementary way with local bulk TAGs. On cooling, the hydrophobic groups in an emulsifier at a dispersed oil droplet interface act as impurities and templates for TAG nuclei, promoting heterogeneous nucleation at the dispersed oil droplet interface. TAG nuclei are present, and TAG crystal formation occurs at the interface of scattered oil droplets [99].

## 3. Conclusions

The stability and structure of emulsion gels involving electrostatic protein-polysaccharide interactions can be influenced by pH alteration, as shown with the EGA and EGB samples. Although no significant differences in proximate analysis, syneresis and thermal stability between all the samples were observed, EGA and EGB showed positive results for other analyses compared to EGC. EGA and EGB had higher pH values, the highest lightness (L*) values, and higher gel strength, hardness and oil binding capacity. The formation of the gel network of the emulsion gels can also be seen in the microstructure images for samples EGA and EGB. The particle size and lipid oxidation values of EGA and EGB were also significantly lower compared to EGC. Furthermore, all the formulated emulsion gels had a reduction in saturated fat and an improved fatty acid profile. DSC melting and cooling curves with single endothermic and exothermic sharp peaks provide clear evidence that the electrostatic protein-polysaccharide interaction did not affect the melting and crystallization characteristics of the emulsion gels. X-ray diffraction showed that the beta prime (β′) polymorphic form was present in all the emulsion gel samples. In conclusion, this study successfully developed emulsion gels, which could be applied as a fat replacer in future research.

## 4. Materials and Methods

### 4.1. Materials and Sample Preparation

Modified corn starch (CLEARAM CH 20) was provided by Roquette (Lestrem, France), and canola oil (NATUREL, Lam Soon, Shah Alam, Malaysia) was purchased from the local supermarket (Lotus’s, IOI City Mall, Putrajaya, Malaysia). Gelatin (HALAGEL) and citric acid were also purchased from the local supermarket (JC Rainbow, Selangor, Malaysia). Food-grade calcium hydroxide was purchased from Modern Pantrist (Eliot, ME, USA).

**Figure 5:**
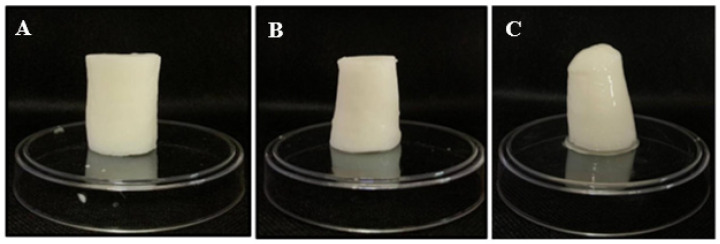
The appearance of emulsion gel A (**A**), emulsion gel B (**B**) and emulsion gel C (**C**).

Three treatments based on the pH were used to prepare the modified corn starch emulsion gels (MCSEG): (1) starch, pH: 4.4, gelatin, pH: 5.4 (EGA); (2) starch, pH: 7.5, gelatin, pH: 4 (EGB); (3) starch, pH: 2.6, gelatin, pH: 9 (EGC). The pH of the dispersion was adjusted with 1 M of citric acid (C_6_H_8_O_7_) and 2 M of calcium hydroxide (Ca (OH)_2_). The emulsion gels were produced in triplicate using the following formulation: 20% modified corn starch, 20% canola oil, 58% water and 2% gelatin. The emulsion gels were prepared by mixing modified corn starch with water for 1 min at high speed (approx. 5600 rpm) using a homogenizer. The gelatin was then added and mixed for 1 min (approx. 5600 rpm). The final mixture was mixed again at approx. 10,000 rpm for 4 min with the gradual addition of canola oil to the mixture. The samples were then heated to 90 °C for 30 min to obtain the emulsion gel. They were stored in a refrigerator at 2 °C for 24 h for further analysis. The procedure to prepare the emulsion gels is described in Table 5. Figure 5 shows the final emulsion gel A, emulsion gel B and emulsion gel C obtained.

### 4.2. Proximate Composition Analysis

The AOAC standard methods [100] were used to perform the analysis. Moisture content was determined using a universal oven (Binder, Tuttlingen, Germany), and the Kjeldahl method was used to determine the total protein (crude protein, N = 6.25) content. Soxhlet-Henkel method was used to assess the fat content and the ash content was quantified by mineralization at 550 °C.

### 4.3. pH Analysis

The pH was determined with a pH meter (PB-10, Sartorius, Gottingen, Germany) [101] by weighing 1 g of the sample was homogenized (1:10 *w/v*) with distilled water using a homogenizer (Diax900, Heidolph, Schwabach, Germany). 

### 4.4. Color Measurement

The emulsion gels were measured objectively by measuring the surface color using a hand-held chromameter (CR400, Minolta Camera Co., Osaka, Japan) with an aperture size of 8 mm. Three samples from each treatment were analyzed, and the average value was determined by taking observations at three locations on the samples. Hunter L* (lightness), a* (redness) and b* (yellowness) values were then determined [102].

### 4.5. Syneresis and Thermal Stability

The method suggested by Verbeken et al. [103] was used with minor adjustments to estimate the syneresis of the gel. In 50 mL graduated centrifuge tubes, emulsion gel dispersions (30 mL) were added, and their masses (m1) were recorded. These dispersions (sol) were stored at 4 °C for 48 h with a cover after being allowed to cool to room temperature for the gel-setting process. Next, these tubes were centrifuged at 5000 rpm (2670 g) for 10 min in a laboratory model centrifuge (Kubota 3740, Osaka, Japan). After centrifugation, the gels, along with the tubes, were weighed (m2) again after discarding the separated water. Syneresis of gel was calculated as (m1 − m2)/m1 and expressed on a percentage basis. Water and fat binding properties were estimated by measuring water and fat loss during heating (as a measure of thermal stability) [104]. After being hermetically sealed, the samples (about 25 g) were placed within tubes and heated in a water bath for 30 min at 70 °C. The separated fat and/or water were then released onto a plate after they were opened and allowed to stand upside down for 50 min. Heating (total) loss (water and fat loss) was measured as % of the initial sample weight. Water loss was determined as % weight loss after heating the total released fluid (fat and water) for 16 h on a stove at 100 °C. Fat loss was calculated as the difference between total loss (measured as % of the initial sample weight) and water loss.

### 4.6. Gel Strength and Hardness

The gel strengths of the emulsion gels were measured using a texture analyzer (TA-XT2, Stable Microsystem System Ltd., Godalming, UK) aided by the software “Texture Expert”. Emulsion gels were cut into cylinders (25 mm in height × 20 mm diameter). The gel strength (g · mm) was measured using a spherical probe of 5 mm p/5S (test speed 1.1 mm · s^−1^, force 10 g, and sample distance 15 mm) [94]. The hardness levels of the emulsion gels were measured using a Texture Analyzer TA.XT Plus (A-XT2i Stable Micro Systems, London), using a stainless steel 4 mm cylindrical probe with the following parameters: pre-test speed 1 mm/s, test speed 3 mm/s, post-test speed 10 mm/s, distance 10 mm, and force 5 g [105].

### 4.7. Particle Size and Zeta Potential

The droplet size distribution and surface-weighted mean diameter (d3.2) of the emulsion gels were measured using laser diffraction (Mastersizer 2000; Malvern Instruments Ltd., Worcestershire, UK) and the zeta (ζ)-potential of emulsion solutions/suspensions were determined via electrophoretic light scattering (Litesizer 500, Anton Paar, Graz, Austria). The refractive indices of water and canola oil were set at 1.33 and 1.47, respectively [106].

### 4.8. Lipid Oxidation

On day 0 and day 13, the thiobarbituric acid-reactive compounds (TBARS) were determined using an extraction method with slight adjustments. A 20% trichloroacetic acid solution (20 mL) was used to homogenise 4 grams of the sample before the slurry was centrifuged at 3000 g (Kubota 3740, Osaka, Japan) for 10 min. Two ml of the supernatant was mixed with an equal volume of freshly prepared 0.1% thiobarbituric acid in a glass test tube and heated in a water bath at 100 °C for 30 min, followed by cooling under tap water. The absorbance of the mixture was measured at 532 nm, and the TBAR values were calculated using a TBA standard curve and expressed in mg malonaldehyde/kg [107].

### 4.9. Oil Binding Capacity (OBC)

For measuring OBC, the method suggested by Palla et al. [108] with slight modifications was used. Weighted emulsion gels were centrifuged at 9170× *g* for 15 min at 20 °C. The tubes were inverted to drain any oil for 5 min. The %OBC was calculated as a function of the released oil after centrifugation as per the equation given below.

%OBC = 100%Released Oil(1)

### 4.10. Microstructure 

The emulsion gel images were recorded via scanning electron microscopy (SEM) (LEO 1455 VPSEM, Cambridge, UK) after the freeze-dried specimens were sputter-coated with gold. The images were obtained with a magnification of 100×, and the most representative micrographs were selected to define microstructural characteristics [101].

### 4.11. X-ray Diffraction

The X-ray diffractometer (SHIMADZU XRD-6000) equipped with a Cu Kα source (λ = 1.54 Å) operated at 30 kV and 30 mA and was used to study the crystalline structure of emulsion gel samples. The study was performed in the region of the diffraction angle (2θ) from 5° to 60° at 2 °C/min. The X-ray diffraction patterns were analyzed with X’pert High Score Plus software.

### 4.12. Fatty Acids Analysis

The fatty acid composition was examined in the emulsion gels following the AOCS methodology [109] in a capillary column gas chromatograph–GC 2010Series GC System (Agilent, Santa Clara, CA, USA), after esterification [110].

### 4.13. Differential Scanning Calorimetry

All emulsion gels were thermally analysed using a Mettler Toledo differential scanning calorimeter (DSC 823 Model, Columbus, OH, USA). Thermal analysis software (STARe software, Version 9.0×, Schwerzenbach, Switzerland) was used to elaborate on the data. 5–15 mg of the sample was placed in an aluminum pan and hermetically sealed, with an empty pan used as a reference. The samples were stored at 4 °C until analysis. Emulsion gels were heated from 0 °C to 100 °C at a rate of 10 °C/min, held at 100 °C for 10 min, and then cooled from 100 °C to −40 °C at a rate of 10 °C/min. Through this analysis, the peak melting temperature (Tm), enthalpy of melting (ΔHm), peak crystallization temperature (Tc), and crystallization enthalpy (ΔHc) were determined [105].

### 4.14. Statistical Analysis

Statistical analysis was performed using Minitab ver. 18.0 (Minitab Inc., State College, PA, USA). Significant differences between the data obtained were determined statistically using one-way analysis of variance (ANOVA) with Tukey’s multiple comparison tests at a 95% confidence level. The values were expressed as a mean ± standard deviation.

## Figures and Tables

**Table 1:** Proximate compositions and pH values of EGA, EGB and EGC.

Samples	EGA	EGB	EGC
Moisture	57.14 ± 1.20 ^a^	58.09 ± 1.02 ^a^	58.95 ± 1.81 ^a^
Ash	2.35 ± 0.29 ^a^	2.53 ± 0.26 ^a^	2.36 ± 0.13 ^a^
Protein	3.28 ± 0.51 ^a^	2.88 ± 0.35 ^a^	2.48 ± 0.17 ^a^
Fat	7.03 ± 0.25 ^a^	6.87 ± 0.99 ^a^	8.33 ± 0.60 ^a^
pH	5.03 ± 0.06 ^a^	4.50 ± 0.00 ^b^	3.63 ± 0.06 ^c^

Values are mean ± standard deviation of three replicates (*n* = 3). Means in the same column that do not share the same lowercase superscript are significantly different (*p* < 0.05).

**Table 2:** Color parameters ((L*) lightness, (a*) redness and (b*) yellowness), thermal stability, gel strength and hardness of EGA, EGB and EGC.

Parameters	EGA	EGB	EGC
L*	91.61 ± 0.24 ^a^	91.54 ± 0.23 ^a^	90.79 ± 0.02 ^b^
a*	−0.84 ± 0.03 ^a^	−1.08 ± 0.08 ^b^	−1.34 ± 0.01 ^c^
b*	8.83 ± 0.04 ^c^	9.63 ± 0.49 ^b^	10.94 ± 0.01 ^a^
Thermal Stability (Total loss)	0.59 ± 0.03 ^a^	0.64 ± 0.19 ^a^	0.87 ± 0.07 ^a^
Thermal Stability (Water loss)	0.04 ± 0.00 ^a^	0.04 ± 0.04 ^a^	0.08 ± 0.04 ^a^
Gel strength	40.16 ± 5.71 ^a^	23.89 ± 2.44 ^b^	18.13 ± 1.09 ^b^
Hardness	1427.11 ± 37.62 ^a^	458.88 ± 75.54 ^b^	136.34 ± 6.56 ^c^

Values are mean ± standard deviation of three replicates (*n* = 3). Means in the same row that do not share the same lowercase superscript are significantly different (*p* < 0.05).

**Table 3:** Particle size, lipid oxidation and oil binding capacity values of EGA, EGB, and EGC.

Parameters	EGA	EGB	EGC
Particle Size Zeta potential	44.53 ± 4.05 ^b^ −6.5 ± 5.19	54.01 ± 1.70 ^b^ +3.5 ± 0.04	68.34 ± 6.04 ^a^ +1.9 ± 1.58
Lipid oxidation (Day 0)	0.28 ± 0.05 ^b^	0.29 ± 0.02 ^b^	0.41 ± 0.06 ^a^
Lipid oxidation (Day 13)	0.34 ± 0.03 ^b^	0.37 ± 0.02 ^b^	0.41 ± 0.06 ^b^
Oil binding capacity	98.04 ± 0.04 ^a^	97.37 ± 1.12 ^a^	91.80 ± 1.23 ^b^

Values are mean ± standard deviation of three replicates (*n* = 3). Means in the same row that do not share the same lowercase superscript are significantly different (*p* < 0.05).

**Table 4:** Fatty acids profile of EGA, EGB and EGC.

Fatty Acids (%)		EGA	EGB	EGC
Saturated fatty acids	Butryic	0.0000	0.0000	0.0000
	Caproic	0.0069	0.0000	0.0000
	Caprylic	0.1144	0.0135	0.0119
	Capric	0.0221	0.0177	0.0169
	Undecanoic	0.0143	0.0000	0.0000
	Lauric	0.0831	0.0717	0.0681
	Tridecanoic	0.0135	0.0000	0.0000
	Myristic	0.4058	0.7792	0.3920
	Pentadecanoic	0.0330	0.0868	0.0443
	Palmitic	6.0921	6.2293	5.5987
	Heptadecanoic	0.1546	0.2059	0.1511
	Stearic	2.5202	2.3468	2.2594
	Arachidic	0.5358	0.4592	0.4648
	Henicosanoic	0.0000	0.0000	0.0000
	Behenic	0.3964	0.1748	0.1809
	Tricosanoic	0.2472	0.0000	0.0000
	Lignoceric	0.0000	0.0000	0.0000
Monounsaturated fatty acids	Myristoleic	0.1820	0.0244	0.0113
	Cis-10-Pentadecenoic	0.0000	0.0000	0.0000
	Palmitoleic	0.5499	1.0102	0.6032
	Cis-10-Heptadecanoic	0.1153	0.1413	0.1097
	Elaidic (Trans)	0.0000	0.0000	0.0000
	Oleic	63.8491	57.6595	58.9921
	Cis-11-Eicosenoic	0.9928	0.9516	0.9385
	Erucic	0.2099	0.0000	0.0000
	Nervonic	0.4363	0.3388	0.2966
Polyunsaturated fatty acids	Linolelaidic (Trans)	0.0000	0.0000	0.0000
	Linoleic (Cis)	17.0443	19.7278	20.2468
	-Linolenic	0.2248	0.3315	0.3336
	a-Linolenic	5.7563	8.6076	8.8154
	Cis-11,14-Eicosadienoic	0.0000	0.0000	0.0000
	Cis-8,11,14-Eicosatrienoic	0.0000	0.0000	0.0000
	Cis-11,14,17-Eicosatrienoic	0.0000	0.0000	0.0000
	Arachidonic	0.0000	0.0000	0.0000
	Cis-5,8,11,14,17- eicosapentaenoic	0.0000	0.4844	0.2484
	Cis-13, 16-Docosadienoic	0.0000	0.0000	0.0000
	Cis-4,7,10,13,16,19-Docosahexaenoic	0.0000	0.3380	0.2162

**Table 5:** pH adjustment values to prepare EGA, EGB and EGC.

Parameters	EGA	EGB	EGC
Starch	4.4	7.5	2.6
Gelatin	5.4	4	9

Emulsion gel A (EGA), Emulsion gel B (EGB) and Emulsion gel C (EGC).

## Data Availability

Data obtained as described.
